# Supplementary material for: Chemical Composition and Antimicrobial Properties of Honey Bee Venom
Source: Molecules. 2023 May 17;28(10):4135. doi: 10.3390/molecules28104135 (PMC10223701; doi:10.3390/molecules28104135)
Supplement: Supplementary file 1 [file molecules-28-04135-s001.zip › molecules-2350294-supplementary.pdf]

Supplement of

Chemical Composition and Antimicrobial Properties of Honey Bee Venom

Valery Isidorov, Adam Zalewski, Grzegorz Zambrowski, Izabela Swiecicka

Correspondence to: Valery A. Isidorov (v.isidorov@pb.edu.pl; Isidorov@uwb.edu.pl)

Table S1. Chemical composition of volatiles from dry and fresh honey bee venom

				Dry bee venom		Stinging apparatus	
Compound	CAS	RI^{Calc}	RI^{Lit}	Dv-1	Dv-2	Fv-1	Fv-2
Ethanol	64-17-5	-	484	1.46	0.50	0.33	0.26
Acetone	67-64-1	-	501	0.59	trace	0.15	2.30
Isobutanal	78-84-2	-	554	1.88	-	-	-
Formic acid	64-18-6	-	519	2.37	-	-	-
2-Butanone	78-93-3	-	598	trace	0.18	1.18	1.56
n-Hexane	110-54-3	600	600	2.62	-	-	-
Ethyl acetate	141-78-6	606	610	0.95	trace	0.54	trace
Acetic acid	64-19-7	612	599	6.60	3.00	7.97	7.83
Isobutanol	78-83-1	624	625	-	2.45	-	-
Isopentanal	590-89-3	648	652	-	0.71	-	-
1-Butanol	71-36-3	664	660	-	2.05	-	-
2-Pentanone	107-87-9	700	702	1.37	trace	0.40	0.73
2-Pentanol	6032-29-7	705	706	-	0.77	0.56	--
Propanoic acid	79-09-4	722	732	-	-	0.28	-
Acetoin	513-86-0	723	723	-	-	0.52	-
2,3-Butanediol-1	5341-95-7	734	737	-	-	0.13	trace
Isopentanol	123-51-3	736	734	0.40	28.9	3.56	5.10
2-Methylbutan-1-ol	137-32-6	739	738	-	-	0.99	-
Pyridine	110-86-1	740	742	0.46	-	0.52	0.71
Toluene	108-88-3	761	761	3.40	1.15	1.93	1.42
Isobutyric acid	79-31-2	762	765	-		0.60	0.91
3-Methyl-2-butenal	107-86-8	782	782	-	0.70	-	-
2,3-Butanediol-2	6982-25-8	782	779	-	-	0.09	0.24
Butanoic acid	107-92-6	798	799	trace	trace	0.59	0.50
n-Octane	111-65-9	800	800	-	0.26	0.14	-
Hexanal	66-25-1	802	802	1.73	-	-	-
3-Methyl-1-pentanol	589-35-5	842	839	-	0.43	-	-
(*E*)-3-Hexen-1-ol	928-97-2	850	852	-	0.73	-	0.23
2,4-Dimethyl-1-heptene	19549-87-2	837	842	0.08	-	-	-
3-Hexen-1-ol	928-96-1	851	855	-	-	0.34	-
Ethyl benzene	100-41-4	857	862	-	-	0.32	-
p-Xylene	106-42-3	866	866	-	-	0.64	-

Isovaleric acid	503-74-2	866	870	0.07	-	0.71	-
1-Hexanol	111-27-3	865	867	trace	8.43	0.46	1.99
2-Methylbutyric acid	116-53-0	868	872	0.04	-	-	-
Isoamyl acetate	123-92-2	874	875	trace	0.13	1.45	4.29
2-Methyl-1-butyl acetate	624-41-9	879	877	-	-	0.47	0.12
3-Heptanone	106-35-4	885	884	-	-	0.41	-
Styrene	100-42-5	889	886	-	-	0.76	-
2-Heptanone	110-43-0	890	888	16.08	0.49	0.76	1.53
n-Nonane	111-84-2	900	900	-	0.39	-	
2-Heptanol	543-49-7	901	899	-	0.21	0.15	0.40
Heptanal	111-71-7	902	902	0.34	-	-	-
3-Hepten-1-ol	2108-05-6		906	-	2.72	0.33	0.58
γ-Butyrolactone.	96-48-0	914	915	-	-	0.32	0.72
β-Thujene	2867-05-2	926	926	-	-	0.89	-
α-Pinene	80-56-8	932	931	1.78	trace	2.06	2.39
3-Methyl-2-butenoic acid?	541-47-9	934		-	1.53	-	-
Ethyl 2-methylvalerate (manzanate)	39255-32-8	940	940	-	-	1.23	1.59
3-Methylheptan-2-one?	2371-19-9	944		-		1.62	1.19
γ-Butyrolactone. 2-methyl-	1679-47-6	953	951	-	-	0.05	0.10
NN	-	953	-	-	-	0.15	
γ-Valerolactone	108-29-2	955	954	trace	-	0.16	trace
Benzaldehyde	100-52-7	958	960	2.62	-	-	0.11
Isoamyl propionate	105-68-0	966	968	trace	0.37	0.06	0.11
β-Pinene	127-91-3	975	973	0.54	0.02	0.47	trace
3,5-Dimethyldihydro-2(3H)-furanone?	5145-01-7	985	-	-	0.42	0.08	-
Phenol	108-95-2	988	990	0.19	-	0.58	0.61
2-Octanone	111-13-7	992	991	-	0.41	0.37	0.73
2-Pentylfuran	3777-69-3	993	990	0.20	-	-	-
Hexanoic acid	142-62-1	1000	998	1.43	0.30	0.76	0.75
n-Decane	124-18-5	1000	1000	-	trace	0.31	0.48
Ethyl hexanoate	123-66-0	1003	1001	trace	--	-	-
Octanal	124-13-0	1003	1003	-	1.04	-	-
3-Carene	13466-78-9	1009	1011	0.36	trace	0.28	0.32
3-Methylbutyl isobutanoate	589-59-3	1010	1006	-	0.76	0.25	0.38
(*E*)-3-Hexenoic acid?	1577-18-0	1015	-	0.68	-	-	-
p-Cymene	99-87-6	1024	1024	-	-	0.24	0.28
Limonene	138-86-3	1029	1028	0.56	0.35	2.90	3.12
1,8-Cineol	470-82-6	1032	1030	0.41	-	-	
2-Ethyl-1-hexanol	104-76-7	1032	1032	-	-	1.84	1.38
Benzyl alcohol	100-51-6	1040	1036	-	-	-	0.24
Salicyl aldehyde	90-02-8	1043	1041	0.44	-	-	-
(*Z*)-β-Ocimene	3338-55-4	1045	1042	0.25	-	-	-
γ-Caprolactone	695-06-7	1056	1056	trace	-	1.71	2.56
1-Phenylethanol	98-85-1	1062	-	-	-	0.12	0.21
2-Acetylpyrrole	1072-83-9	1064	1062	trace	-	-	-

Acetophenone	98-86-2	1064	1066	0.33	trace	0.43	0.58
(Z)-3-Octen-1-ol	18409-17-1	1069	1067	-	0.23	-	0.75
1-Octanol	111-87-5	1070	1072	-	1.92	-	-
Dihydromyrcenol	18479-58-8	1073	1072	-	-	2.37	3.08
1-Nonen-3-ol	21964-44-3		1080	-	0.29	-	-
o-Guaiacol	90-05-1	1089	1091	-	-	0.52	-
3-Nonanone	925-78-0		1090	-	-	1.58	0.39
2-Nonanone	821-55-6	1093	1089	1.99	1.57	6.61	10.94
Methyl benzoate	93-58-3	1095	1094	1.88	-	-	-
n-Undecane	1120-21-4	1100	1100	trace	trace	-	-
Tetrahydrolinalool?	78-69-3	1097	1098	-	-	0.87	-
2-Nonanol	628-99-9	1103	1102	-	0.57	5.79	-
Nonanal	124-19-6	1105	1005	0.71			-
Isopentyl isovalerate (apple oil)	659-70-1	1106	-	-	1.83	0.45	0.83
2-Phenylethanol	60-12-8	1112	1112	-	trace	1.39	0.82
Methyl octanoate	111-11-5	1126	1126	0.96	-	-	-
NN	-	1132	-	-	-	0.29	0.28
Camphor	76-22-2	1144	1146	-	-	0.28	0.33
γ-Heptalactone	105-21-5	1155	1155	-	trace	2.05	2.05
2-Methylundecane	7045-71-8	1161	1164	-	-	0.08	0.14
Borneol	507-70-0	1166	1168	-	-	0.32	0.37
3-Methylundecane	1002-43-3	1171	1172	-	-	0.13	0.12
NN	-	1175	-	-	-	0.16	-
Octanoic acid	124-07-2	1180	1182	20.72	-	0.93	0.50
Benzoic acid	65-85-0	1183	1180	-	-	0.76	-
Isoamyl 3-methyl-2-butenoate	N/A	1185		-	2.67	1.15	0.14
α-Terpineol	98-55-5	1191	1190	-	-	0.94	0.93
Creosol (*p*-methylguaiacol)	93-51-6	1193	1192	-	-	1.22	1.29
Ethyl octanoate	106-32-1	1200	1198	10.57	trace	-	-
n-Dodecane	112-40-3	1200	1200	-	trace	2.00	0.26
Octyl acetate	112-14-1	1213	1214	trace	0.50	0.14	0.61
3-Octenoic acid	1577-19-1	1214	-	0.50	-	0.51	-
2-Octenoic acid	1871-67-6	1233	1245	0.50	-	0.51	0.15
Acetate		1239	-	-	-	0.49	1.46
NN		1243	-	-	-	0.91	0.56
NN		1245	-	-	-	0.22	0.09
NN		1251	-	-	-	0.27	0.25
NN		1255	-	-	-	0.66	0.49
Octanolide (γ-octalactone)	104-50-7	1259	1259	0.30		1.09	0.95
(E2)-Decenal	3913-81-3	1263	1262	-	-	0.30	0.23
NN	-		1265	-	-	0.17	-
(2E)-Decen-1-ol	18409-18-2	1273	1271	-	-	0.55	0.79
1-Decanol	112-30-1	1273	1274	-	-	0.17	0.28
NN	-	1278	-	-	-	0.14	-
p-Ethylguaiacol	2785-89-9	1284	1280	-	-	0.37	0.37
NN	-	1284	-	-	-	0.62	0.46

Bornyl acetate	76-49-3	1287	1286	-	-	0.64	0.38
3-Undecanone?	2216-87-7	1283	1290		0.23	0.11	0.09
Cyclohexanol acetate, *cis*-2-*tert*-butyl-.	20298-69-5	1295	1293	-	-	0.57	0.33
2-Undecanone	53452-70-3	1294	1295	-	-	0.57	0.76
NN	-		1297	-	-	0.39	-
n-Tridecane	629-50-5	1300	1300	0.33	0.19	0.16	0.28
2-Undecanol	1653-30-1	1305	1303	-	-	0.42	0.40
NN	-	-	1314	-	-	0.08	-
Ester	-	-	1325	-	-	0.12	-
γ-Nonalactone	104-61-0	1362	1363	-	-	0.08	-
Decanoic acid + C15H24	-	1377	1374	-	-	0.15	0.12
Ester (70,57,71,43,83….213)	-	1396		-	-	0.08	0.23
n-Tetradecane	629-59-4	1400	1400	-	-	0.10	0.11
(*E*2)-Decenyl acetate	2497-23-6	1406	1411	-	-	0.17	0.49
β-Caryophyllene	87-44-5	1418	1417	-	0.21	0.06	0.17
Isoamyl benzoate	94-46-2	1438	1437	-	0.23	0.16	0.29
Isoamyl octanoate	2035-99-6	1450	1448	-	trace	0.08	0.08
Geranyl acetone	3796-70-6	1454	1454	-	-	trace	-
4,11-Dimethyltetradecanoate	55045-12-0	1462	1462	-	0.26	0.08	-
NN	-	1480	-	-	-	0.07	-
NN	-	1483	-	-	-	0.06	-
1-Pentadecene	13360-61-7	1493	1492	-	16.99	0.17	0.10
n-Pentadecane	629-62-9	1500	1500	-	0.49	1.44	1.36
γ-Cadinene	N/A	1517	1513	-	-	0.07	0.11
(*E*)-Calamenene	73209-42-4	1524	1522	-	-	0.07	0.09
7-Hexadecene?	N/A	1576	1579	-	-	0.06	trace
Hexadecane	544-76-3	1600	1600	-	-	0.15	0.09
NN	-	1662	-	-	-	0.08	-
NN	-	1669	-	-	-	0.18	
8-Heptadecene?	54290-12-9	1678	1680	-	0.30	3.18	0.09
n-Heptadecane	629-78-7	1700	1700	-	0.18	1.47	1.95
NN	-	1721	-	-	-	0.09	-
NN	-	1733	-	-	-	0.06	-
NN	-	1738	-	-	-	0.25	-
Ester (nonyl nonanoleate?)	-	1750	-	-	-	0.06	-
Ester	-	1763	-	-	-	0.18	-
Ester	-	1771	-	-	-	0.12	-
Octadec-7-ene	N/A	1774	1776	-	-	0.13	-
Ester	-	1783	-	-	-	0.48	-
Ester	-	1801	-	-	-	0.40	-
isopropyl tetradecanoate?	110-27-0	1828	1827	-	-	0.60	1.09
Ester	-	1830	-	-	-	0.14	-
9-Nonadecene?	31035-07-1	1874	1877	-	0.45	5.51	2.87
Nonadecene, isomer	-	1878	-	-	0.10	0.16	-
n-Nonadecane	629-92-5	1900	1900	-	-	0.21	0.09

Table S2. Relative chemical composition (% TIC) of ether extracts from dry preparation of bee venom (Dv-1 & Dv-2) and fresh venom (Fv-1 & Fv-2) of honey bees

Compound	CAS	RI^{Calc}	RI^{Lit}	Dry venom		Fresh venom	
				Dv-1	Dv-2	Fv-1	Fv-2
Isobutyric acid, TMS	16844-98-7	831	832	-	-	0.05	-
3-Methylbutyric acid, TMS	55557-13-6	932	932	-	-	0.93	0.16
Ethyl amine, di-TMS	2477-39-6	966	966	-	8.44	1.07	2.57
Carbodiimide, TMS	1000-70-0	968	966	0.52	1.23	-	-
Boric acid, tri-TMS	4325-85-3	1001	1002	-	-	0.30	-
3-Methyl-3-butenoid acid,TMS	25436-25-3	1007	1008	0.06	-	-	0.47
1,2-Propanediol, di-TMS	17887-27-3	1009	1010	-	-	0.03	-
Crotonic acid, TMS	25436-25-3	1013	1012	-	0.06	0.43	-
2,3-Butanediol, di-TMS, isomer 1	53274-85-4	1043	1043	-	-	0.23	-
2,3-Butanediol, di-TMS, isomer 2	N/A	1050	1049	-	-	0.47	-
Phenol, TMS	1529-17-5	1054	1055	-	-	0.03	-
1,3-Propanediol, di-TMS?	17887-80-8	1059	1050	-	-	0.04	-
Lactic acid, di-TMS	17596-96-2	1074	1073	0.05	0.05	0.48	-
Benzyl alcohol, TMS	14642-79-6	1156	1156	-	-	0.05	-
α-Hydroxyisovaleric acid, di-TMS	55124-92-0	1178	1175	-	-	0.07	-
1-Octanol	14246-16-3	1182	1184	-	-	0.03	0.03
2,5-Hexanediol, di-TMS?	66956-94-3	1217	-	-	-	0.07	-
3-Hydroxyisovaleric acid, di-TMS	55124-90-8	1219	1216	-	-	0.05	-
2-Phenylethanol, TMS	14629-58-4	1228	1228	-	-	trace	-
Benzoic acid, TMS	2078-12-8	1248	1249	-	-	-	0.30
2-Hydroxy-3-methylvaleric acid, di-TMS	54890-09-4	1254	1253	-	-	0.13	-
Octanoic acid, TMS	55494-06-9	1269	1266	-	-	0.10	0.03
H3PO4, tri-TMS	10497-05-9	1285	1289	0.44	0.99	-	-
Glycerol, tri-TMS	6787-10-6	1289	1293	0.36	0.13	0.07	-
Benzeneacetic acid, TMS	2078-18-4	1301	1303	-	-	0.04	0.01

Thymol, TMS	55012-80-1	1318	1321	-	-	0.04	-
2-Octenoic acid, TMS?	N/A	1321	1313	-	-	0.08	0.02
Succinic acid, di-TMS	40309-57-7	1325	1324	-	trace	0.06	-
Uracil, di-TMS	10457-14-4	1351	1354	-	-	0.04	-
Nonanoic acid, TMS	82326-11-2	1366	1366	-	trace	-	
Citronellic acid, TMS?	N/A	1394	-	-	-	0.16	-
NN	-	1410	-	-	-	0.05	-
NN	-	141		-	-	0.24	-
NN	-	1463	-	-	-	0.15	-
3-Hydroxyoctanoic acid, di-TMS	136788-83-5	1488	1488	-	-	0.03	-
1-Pentadecene	13360-61-7	1492	1492	-	-	-	0.08
Pyroglutamic acid, di-TMS	30274-77-2	1533	1530	-	0.06	-	-
Tetrose, TMS?	N/A	1585	-	0.10	-	-	-
4-Hydroxybenzoic acid, di-TMS	2078-13-9	1632	1636	-	-	0.28	0.22
2-Hydroxydecanoic acid, di-TMS	N/A	1653	1651	-	-	0.02	-
8-Heptadecene?	2579-04-6	1675	-	-	-	-	0.01
n-Heptadecane	629-78-7	1700	1700	-	-	trace	-
Suberic acid, di-TMS	43199-48-0	1707	1711	-	-	0.12	-
NN	-	1715	-	-	-	0.09	-
Vanillic acid, di-TMS	2078-15-1	1773	1776	-	-	0.09	-
(E,Z)-Farnesol, TMS	N/A	1813			-	0.05	-
Hexafuranose, TMS	-	1802		0.12	-	-	-
Neophytadiene 1	N/A	1841	1840	0.05	-	-	-
Tetradecanoic acid, TMS	18603-17-3	1852	1853	-	-	0.13	-
Citric acid, TMS	14330-97-3	1855	1852	1.88	3.79	-	-
9-Nonadecene	31035-07-1	1876	1880	0.13	0.10	0.78	0.55
n-Nonadecane	629-92-5	1902	1900	0.20	0.15	-	0.03
Sebacic acid, di-TMS	18408-42-9	1905	1906	-	-	0.45	-
Vanillypropionic acid, di-TMS	56051-49-1	1907	1908	-	-	-	0.05
Pentadecanoic acid, TMS	74367-22-9	1954	1954	-	-	-	0.01

Indole-3-acetic acid, di-TMS	56114-66-0	1972	1977	-	-	0.06	-
Gallic acid, TMS	2078-17-3	1986	1985	1.52	0.88	-	-
(Z)-9-Hexadecenoic acid, TMS?	N/A	2025	-	-	-	0.32	-
Palmitelaidic acid, TMS	82326-15-6	2028	2030	-	-	0.39	0.09
β-Glucopyranose, TMS	3327-61-5	2031	2032	0.04	-	-	-
Hexadecanoic acid, TMS	55520-89-3	2053	2052	0.34	0.29	2.40	4.93
(Z)-9-Heneicosene	39836-21-0	2074	2071	0.05	trace	-	0.01
n-Heneicosane	629-94-7	2100	2100	0.39	0.32	0.12	0.17
(Z)-10-Heptadecenoic acid, TMS	N/A	2126	-	-	-	0.10	0.01
Oleyl alcohol, TMS	78695-25-7	2137	2136	0.10	0.09	0.29	0.23
Heptadecanoic acid, TMS	55517-58-3	2150	2149	-	-	-	0.04
1-Octadecanol, TMS	18748-98-6	2164	2164	0.08	0.06	0.27	0.12
NN	-	2199	-	-	-	0.23	-
n-Docosane	629-97-0	2200	2200	0.07	0.06	-	0.03
NN	-	2211	-	-	-	0.18	-
Linoleic acid, TMS	56259-07-5	2215	2215	0.04	trace	2.12	1.94
Oleic acid, TMS	21556-26-3	2222	2222	0.80	0.65	12.15	20.14
Octadecanoic acid	18748-91-9	2250	2250	0.06	0.06	3.18	2.76
NN	-	2264	-	-	-	-	0.22
(Z)-9-Tricosene	27519-02-4	2274	2274	0.42	0.36	0.20	0.16
7-Tricosene	N/A	2280	2279	-	-	-	0.02
n-Tricosane	638-67-5	2300	2300	2.15	1.70	0.95	1.55
(10Z)-Nonadecenoic acid, TMS?	N/A	2318	-	-	-	0.06	-
(9Z)-Eicosen-1-ol, TMS	N/A	2337	-	14.75	11.71	12.40	16.16
10-Eicosen-1-ol, TMS?	N/A	2342	-	0.17	0.12	-	0.30
Nonadecanoic acid, TMS	74367-35-4	2349	2352	-	-	0.10	-
1-Eicosanol, TMS	N/A	2361	2360	0.06	0.16	0.87	0.39
NN (43,55,82,96, 81,278...)	-	2385	-	1.30	1.04	0.16	0.2
n-Tetracosane	646-31-1	2400	2400	0.17	0.13	trace	0.44

11,14-Eicosadienoic acid, TMS?	N/A	2414	-	-	-	0.06	-
11-Eicosenoic acid, TMS?	N/A	2420	-	-	-	0.90	0.28
(Z13)_Eicosenoic acid, TMS?	N/A	2422	-	-	-	-	0.29
Eicosanoic acid, TMS	55530-70-6	2447	2447	-	-	0.26	0.29
9-Pentacosene	N/A	2475	2471	0.67	0.51	0.65	0.38
7-Pentacosene	N/A	2482	-	0.09	0.04	0.12	-
n-Pentacosane	629-99-2	2503	2500	3.41	2.63	2.16	1.75
1-O-Hexadecylglycerol, di-TMS	N/A	2507	-	0.09	0.07	-	
12-Docosenol, TMS?	N/A	2532	-	0.20	0.15	0.46	0.24
11-Methylpentacosane	15689-71-1	2530	2529	-	0.06	-	0.18
1-Docosanol, TMS	42449-18-3	2558	2557	0.06	-	0.33	0.57
NN	-	2562	-	-	-	0.05	-
NN (57...337...)	-	2574	-	0.07	-	-	-
n-Hexacosane	630-01-3	2600	2600	0.20	0.17	0.20	0.57
NN	-	2634	-	-	-	0.06	-
Docosanoic acid, TMS	74367-36-5	2645	2646	-	-	0.38	0.21
2-Methylhexacosane	N/A	2661	2663	-	-	0.05	-
9-Heptacosene	N/A	2676	-	0.38	0.29	0.50	0.20
7-Heptacosene	N/A	2680	2682	trace	0.08	0.18	-
n-Heptacosane	593-49-7	2702	2700	11.09	8.55	3.55	3.39
Sucrose, TMS	19159-25-2	2717	2714	0.16	0.31	-	-
13-Methylheptacosane	15689-72-2	2732	2731	0.45	0.34	0.21	0.89
NN		2744	-	-	-	0.10	0.22
1-Tetracosanol, TMS	N/A	2755	2754	0.19	0.16	0.47	0.35
n-Octacosane	630-02-4	2800	2800	0.16	0.13	0.26	0.18
Trehalose, octa-TMS	N/A	2816	2815	-	0.08	-	-
Squalene	111-02-4	2829	2828	0.10	trace	0.07	0.38
Tetracosanoic acid	74367-37-6	2844	2845	-	-	1.24	0.18
2-Methyloctacosane	1560-98-1	2861	2861	-	-	0.09	-

Glyceride (205,73,147,117,57...501?)	-	2871	-	1.86	1.43	-	-
9-Nonacosene	N/A	2875	2875	0.15	0.22	0.48	0.14
7-Nonacosene	N/A	2882	2882	1.22	0.94	0.76	0.18
1-Eicosylglycerol, di-TMS	N/A	2896	2892	3.16	2.43	0.95	0.58
n-Nonacosane	630-03-5	2904	2900	3.28	2.46	3.35	2.31
δ-Tocopherol, TMS	52704-11-7	2913	2915	0.09	-	-	-
NN	-	2933		0.34	0.19	0.32	-
1-Hexacosanol, TMS	391679-86-0	2952	2951	0.23	0.17	0.37	0.33
n-Triacontane	638-68-6	3000	3000	0.12	0.09	0.13	0.08
NN	-	3055	-	0.37	0.30	0.12	0.28
NN (55...432)	-	3061	-	0.48	0.39	1.08	-
NN (55....)	-	3070	-	0.47	0.37	0.58	0.46
9-Hentriacontene	N/A	3078	3078	4.42	3.46	3.28	2.45
7-Hentriacontene,	N/A	3085	3082	3.34	2.62	2.40	2.30
n-Hentriacontane	638-68-6	3103	3100	2.23	1.76	2.40	2.31
Kaempherol, tetra-TMS	N/A	3113	3114	-	-	0.14	-
13-Methyltriacontane	N/A	3130	3132	-	0.13	-	0.55
1-Octacosanol, TMS	959269-04-6	3149	3148	-	-	-	0.29
NN (82...502)	-	3153		0.69	0.61	-	-
9-Dotriacontene	N/A	3175	3175	-	0.16	-	0.26
n-Dotriacontane	544-85-4	3200	3200	-	-	trace	0.03
Octacosanoic acid, TMS	1206693-36-8	3244	3241	-	-	0.60	-
24-Methylenecholesterol, TMS?	N/A	3252	3244	-	-	1.94	3.36
NN	-	3255	-	-	1.10	-	1.43
Tritriacontadiene C33H64?	-	3257	-	1.34	-	2.46	2.41
9-Tritriacontene	N/A	3273		1.13	0.45	0.58	4.77
7-Tritriacontene	N/A	3282	-	9.20	0.89	3.70	trace
Triacontene, isomer	-	3286	-	0.63	0.46	-	-
n-Tritriacontane	630-05-7	330	3300	0.41	0.30	0.33	0.34

Ellagic acid, TMS	N/A	3330	3328	0.24	0.17	-	-
β-Sitosterol, TMS	2625-46-9	3346	3345	-	-	-	1.29
1-Triacontanol, TMS	N/A	3347	3347	0.51	0.40	1.05	-
NN (aldehyde?)	-	3350	-	-	0.33	0.42	-
Avenasterol, TMS	55527-94-1	3365	3360	0.23	0.10	0.94	1.65
5α-Stigmast-7-en-3β-ol, TMS?	N/A	3406	-	-	-	0.04	0.21
NN	-	3420	-	-	-	0.08	-
Triacontanoic acid, TMS	1206693-37-9	3440	3440	-	-	0.65	0.04
NN (96,82,55,83…488)	-	3452	-	-	0.14	0.65	0.22
NN	-	3463	-	-	-	0.15	0.27
NN	-	3470	-	-	-	0.08	-
9-Pentatriacontene	-	3474		-	0.14	-	0.26
7-Pentatriacontene	-	3477	-	-	0.08	-	0.18
1-Dotriacontanol, TMS	N/A	3545	3542	0.20	0.18	0.40	0.23
Aldehyde?	-	3550	-	0.28	0.24	-	--
Ester (palmitate)	-		-	2.59		-	-
Unsaturated acid, TMS C35H70O2Si	-	3622	-	-	-	0.04	-
Dotriacontanoic acid, TMS	N/A	3640	-3641	-	-	0.30	-
Alkene C37H72?	-	3737	-	-	-	0.10	-
1-Tetratriacontanol, TMS	N/A	3743	3743	-	-	0.09	-
Stearyl linoleate?	N/A	3753	-	-	2.67	0.62	0.22
Eicosyl palmitate	22413-01-0	3770	-	-	-	0.14	-
Unsaturated acid, TMS C37H74O2Si	-	3823	-	-	-	0.04	-
Tetratriacontanoic acid, TMS	N/A	3840	3838	-	-	0.38	-
Ester (oleate)	-	3937	-	13.18	17.42	1.89	1.37
Ester (oleate)	-	3954		1.54	1.51	0.92	0.56
Hexatriacontanoic acid, TMS	N/A	4040	-	-	-	0.06	-
Tetracosyl palmitate	N/A	4173	-	-	0.39	3.42	1.20

Table S3. Chemical composition of methanol extracts of dried bee venom (Dv-1 and Dv-22) and the fresh honey bee venom (Fv-1 and Fv-2)

				Dry venom		Fresh venom	
Compounds	CAS	RI^{Calc}	RI^{Lit}	Dv-1	Dv-2	Fv-1	Fv-2
Isovaleric acid, di-TMS	55557-13-6	932	932	-	-	-	0.02
Alanine, mono-OTMS	N/A	933	-	-	-	0.21	0.18
Ethylamine, di-TMS	2477-33-6	964	959	-	1.75	0.88	1.52
Glycine, methyl ester, mono-TMS	25688-7-6	966	971	-	0.04	-	-
Carbodiimide, di-TMS	1000-70-0	966	965	0.30	0.17	0.49	0.60
Ethylene glycol, di-TMS	7381-30-8	991	993	-	0.08	trace	0.08
3,3-Dimethylacrylic acid, TMS	25436-25-3	1007	-	-	-	-	0.13
3-Methyl-3-butenoic acid, TMS	25436-25-3	1008	-	0.10	0.10	-	-
1,2-Propanediol, di-TMS	17887-27-3	1009	1010	-	-	-0.05	-
Ethanolamine, N,O-di-TMS	17165-52-5	1021	-	-	-	0.06	trace
3-Methylpyridine, mono-TMS	41571-88-4	1036	-	-	0.02	-	0.50
Carbamate, tri-TMS	N/A	1040	-	-	-	-	0,15
2,3-Butanediol, di-TMS, isomer-1	53274-85-4	1043	1043	-	-	0.11	0.07
2,3-Butanediol, di-TMS, isomer-2	53274-85-4	1050	1049	-	-	0.33	0.07
Phenol, mono-TMS	1529-17-5	1054	1055	-	-	-	-
1,3-Propanediol, di-TMS?	17887-80-8	1059	1050	-	-	0.08	-
Lactic acid, di-TMS	17596-96-2	1071	1073	0.49	0.22	4.00	0.38
Glycolic acid, tri-TMS	33581-77-0	1082	1083	0.11	0.05	-	0.10
Alanine, N,O-di-TMS	2899-44-7	1112	1114	0.91	0.07	3.52	2.62
Glycine, N,O-di-TMS	7364-42-3	1128	1128	-	--	0.60	0.44
Sarcosine, N,O-di-TMS	7364-43-4	1151	1150	-	-	0.49	0.27
β-Lactic acid, di-TMS	55162-32-8	1157	1155	-	-	0.11	0.20
Benzyl alcohol, TMS	14642-79-6	1156	1156	-	trace	-	-
3-Hydroxybutyric acid, di-TMS	55133-94-3	1167	1167	-	-	0.06	trace
Proline, mono-OTMS	N/A	1173	1174	-	-	-	0.10
α-Hydroxyisovaleric acid, di-TMS	55124-92-0	1178	1175	-	-	0.10	-
1-Octanol, mono-TMS	14246-16-3	1182	1184	-	-	-	-
Methylphosphoric acid, di-TMS	18291-81-1	1190	1190	-	-	trace	trace
β-Alanine, N,O-di-TMS	1789-86-0	1199	1198	-	-	0.51	0.28
2,5-Hexanediol, di-TMS?	66956-94-3	1217	-	-	-	-	-
3-Hydroxyisovaleric acid, di-TMS	55142-90-8	1220	1220	0.30	0.04	0.12	0.24
Valine, N,O-di-TMS	7364-44-5	1228	1227	trace	0.02	1.85	0.87
Benzoic acid, mono-TMS	2078-12-8	1250	1248	trace	0.05	0.09	0.08
4-Methylpentanoic acid, 2-hydroxy-, di-TMS	54890-08-3	1250	1252	0.10	0.06	0.09	-
3-Methylpentanoic acid, 2-hydroxy, di-TMS	54890-09-4	1253	1253	-	0.04	0.05	-
Urea, di-TMS	18297-63-7	1254	1254	-	0.04	0.15	
Ethanolamine, N,N,O-tri-TMS	5630-81-9	1278	1275	0.12	0.18	0.34	0.33
Leucine, N,O-di-TMS	15984-97-1	1287	1284	trace	0.02	2.87	1.00
H3PO4, tri-TMS	10497-05-9	1290	1289	11.54	6.89	2.48	5.13
Glycerol, tri-TMS	6787-10-6	1294	1293	0.42	1.15	2.55	2.24
Proline, di-TMS	7364-47-8	1307	1303	0.77	0.45	4.30	4.88
Glycine, tri-TMS	5630-82-0	1318	1314	0.14	0.04	0.52	0.32

Thymol, mono-TMS	55012-80-1	1318	1321	-	-	-	-
2-Octenoic acid, mono-TMS?	N/A	1321	1313	-	-	-	-
1-Amino-2-propanol, tri-TMS	N/A	1324	1324	trace	0.06	-	-
Succinic acid, di-TMS	40309-57-7	1326	1324	0.67	0.16	1.04	0.52
Glyceric acid, tri-TMS	38191-87-6	1352	1348	0.62	0.04	-	-
Uracil, di-TMS	10457-14-4	1353	1354	-	-	0.40	0.43
Itaconic acid, di-TMS	55495-04-7	1355	1358	trace	0.03	0.04	trace
Fumaric acid, di-TMS	17962-03-7	1357	1357	-	0.08	-	-
NN (174,73,248.147,175)	-	1365	-	0.21	0.34	-	0.11
Pipecolic acid, TMS	55255-44-2	1365	1366	-	-	trace	trace
Serine, N,O,O-tri-TMS	64625-17-8	1380	1374	trace	0.02	0.99	0.66
Threonine, tri-TMS	64569-35-3	1405	1406	-	-	1.10	0.60
Hydroquinone, di-TMS	2117-24-0	1410	1410	-	0.03	--	-
Citronellic (3,6-dimethyl-6-octenoic) acid?, TMS	N/A	1394	-	-	-	-	-
2,4-Dihydroxybutanoic acid, tri-TMS	55191-52-1	1435	1432	trace	-	-	-
β-Alanine, N,N,O-tri-TMS	55255-77-1	1440	1438	0.10	0.15	1.08	0.65
NN (174,73,147,175,248)	-	1454	-	-	0.06	-	-
NN (73,273,147,182)	-	1460	-	-	0.03	-	-
3,4-Dihydroxybutanoic acid, tri-TMS	55191-53-2	1457	1455	0.32	-	-	-
NN		1460	-	-	0.03	-	0.13
Homoserine, N,O,O-tri-TMS	1177129-58-6	1463	1461	-	-	0.18	trace
2-Aminomalonic acid, tri-TMS	959080-51-4	1489	1479	-	-	-	trace
Malic acid	38166-11-9	1512	1510	0.34	0.23	0.14	0.24
Adipic acid, di	18105-31-2	1517	1517	-	-	0.20	trace
Pentonic acid, 2-deoxy-3,5-dihydroxy-, γ-lactone	74742-34-0	1522	1518	0.21	-	-	-
5-Oxoproline	30274-77-2	1535	1533	2.05	-	0.10	1.28
Threitol	32381-52-5	1534	1535	-	0.04	0.52	0.24
4-Aminoburyric acid (GABA)	39508-23-1	1540	1541	-	-	trace	0.27
Hydroxyproline	55429-66-8	1542	1543	-	-	0.09	0.92
Aspartic acid, N,N,O-tri-TMS	55268-53-6	1544	1537	trace	-	--	-
5-Methylcytosine, N,O-di-TMS	32865-88-6	1547	1542	trace	-	-	-
GABA (4-aminobutyric acid), N,O-di-TMS	39508-23-1	1544	1541	-	trace	-	-
Phenylalanine, mono-OTMS	2899-42-5	1547	1547	-	-	-	0.21
Cysteine, N,O,S-tri-TMS	7364-50-3	1547	1548	-	trace	-	-
NN (73,174,147,175,292)	-	1577	-	-	0.06	-	-
Threonic acid, tetra-TMS	N/A	1577	1575	0.16	-	-	-
α-Hydroxyglutaric acid	55530-62-6	1589	1588	-	-	-0.37	0.09
β-Phenyllactic acid, di-TMS	27750-45-4	1593	1596	-	-	0.12	-
Erythronic acid, tetra-TMS	38191-88-7	1595	1597	0.14	0.04	-	trace
Histamine, N,N'-di-TMS	N/A	1624	1616	-	0.28	-	-
4-Hydroxybenzoic acid, di-TMS	2078-13-9	1636	1636	0.12	0.11	-	0.27
5-Aminovaleric acid, tri-TMS	55191-54-3	1637	1639	-	-	-	0.27
Glutamine, N,O,O-tri-TMS	15985-07-6	1643	1642	trace	0.10	1.90	1.65
Arabinoic acid	32384-55-7	1649	1650	-	-	-	0.18
NN (240,73,170,97,70)	-	1652	-	0.49	0.06	-	-
NN (N-acetylputrescine like), TMS	-	1657	-	-	0.11	-	-

Homocysteine, N,O,S-tri -TMS	N/A	1679	1676	-	-	-	0.19
2-Deoxyribonic acid, tetra-TMS	N/A	1682	1682	-	-	0.11	-
Suberic acid, di-TMS	43199-48-0	1707	1711	-	-	trace	-
NN	-	1710		-	-	0.12	0.35
NN (73,147,103,246,318)	-	1717	-	-	0.01	0.09	-
Isocitric lactone, di-TMS	65143-62-6	1718	1713	-	0.02	-	-
Pentitol, penta-TMS	-	1732		-	-	0.11	0.18
n-Heptadecane	629-78-7	1700	1700	-	-	trace	-
6-Aminohexanoic acid, tri-TMS	25688-76-0	1736	1739	-	0.03	-	-
Putrescine, tetra-	39772-63-9	1751	1749	0.72	0.42	1.54	1.49
Xylitol, penta-TMS	14199-72-5	1758	1750	0.28	-	0.18	0.47
NN (73,147,174,273,215)	-	1759	-	-	0.24	-	-
Ribitol, penta-TMS	323811-53-6	1762	1763	0.10	0.22	0.47	0.44
Arabinitol, penta-TMS	25138-28-7	1767	1772	-	0.04	-	-
Vanillic acid, di-TMS	2078-15-1	1773	1776	-	-	-	-
(*E*)-Aconitic acid, tri-TMS	55530-72-8	1774	-	-	0.09	-	-
N-Acetylputrescine, di-TMS	N/A	1779	1774	-	0.21	-	-
NN (73,273,147,75,89)	-	1790	-	-	0.16	-	-
Glutamine, N,N,O-tri-TMS	70591-28-5	1793	1796	-	-	0.74	0.32
α-Glycerophosphate	31038-11-6	1796	1798	0.69	0.28	2.33	3.31
NN	-	1803	-	-	-	-	0.20
Hypoxanthine, N,O-di-TMS	17962-89-9	1816	1812	-	-	0.75	0.33
Xylonic acid, penta-TMS?	N/A	1822	-	-	0.02	trace	0.70
Cyclohexanepentol, penta-TMS	N114656-62-1	1834	1832	-	0.02	0.22	-
α-Fructofuranose, penta-TMS	N/A	1843	1844	0.98	0.66	1.75	2.49
Cadaverine, tetra-TMS	65898-76-2	1848	-	0.33	1.00	-	-
β-Fructofuranose, penta-TMS	N/A	1851	1854	0.65	13.16	2.83	1.74
Tetradecanoic acid, mono-TMS	18603-17-3	1852	1853	-	-	-	-
Isocitric acid, TMS	14330-97-3	1860	1862	29.51	27.06	0.09	3.00
Lysine, N',N',O-tri	24595-69-5	1864	1860	-	0.02	-	1.05
Pinitol, penta-TMS	N/A	1870	1871	0.38	-	0.19	0.18
9-Nonadecene	31035-07-1	1872	1880	-	-	-	-
Adenine, N,N'-di-TMS	17995-04-9	1875	1874	-	-	-	0.29
Histamine, tri-TMS	N/A	1887	1886	5.74	18.25	1.81	0.35
β-Fructopyranose, penta-TMS	N/A	1887		-	-	0.24	trace
β-Glucofuranose, penta-TMS	N/A	1888		-	-	0.78	0.91
α-Sorbopyranose, penta-TMS	30645-02-4	1904	1903	-	-	0.19	-
Sebacic acid, di-TMS	18408-42-9	1905	1906	-	-	trace	-
Vanilpropionic acid, di-TMS	56051-49-1	1908	1905	-	-	0.08	0.15
Glycylglycine, N,N,O,O-tetra-TMS	6000-14-2	1912	-	-	-	-	trace
Gluconic acid, d-lactone	55515-29-2	1920	1919	-	0.53	0.18-	0.35
Phenyl ethanolamine, N,O,O-tri-TMS	68595-84-6	1924	1921	0.21	-	-	-
α-Glucopyranose, penta-TMS	3327-61-5	1934	1932	0.82	3.03	5.41	7.15
β-Mannopyranose, penta-TMS	55529-69-6	1943	1943	-	0.04	0.13	0.35
Lysine, tetra-TMS	55429-07-7	1946	1944	-	0.02	0.49	-
Noadrenaline, tetra-TMS	68595-65-3	1951	-	-	0.50	0.16	-

Aminocarbohydrate, TMS (203,73,204,147,131,355...539)	-	1951	-	0.13	-	-	-
Aminocarbohydrate, TMS (203,73,204,147,131,355...539)	-	1957	-	1.17	trace	--	-
Aminocarbohydrate, TMS (131,73,147,132,75…539)	-	1964	-	0.24	0.03	-	-
Carbohydrate derivative, TMS	-	1965	-	-	-	-	0.35
Mannitol, hexa-TMS	14317-07-8	1968	1972	0.04	0.59	0.77	1.34
Indole-3-acetic acid, di-TMS	56114-66-0	1972	1977	-	-	-	-
Glucitol, hexa-TMS	14199-80-5	1981	-	-	0.24	2.00	2.76
Gallic acid, tetra-TMS	2078-17-3	1986	1985	0.30	0.23	-	-
hiro-Inositol, hexa-TMS	29412-25-7	1996	1997	-	2.02	-	-
NN	-	1999-	-	-	-	0.90	1.22
Galactosamine, penta-TMS	N/A	2003	2005	0.17	-	-	-
β-Glucopyranose, penta-TMS	2775-90-8	2032	2032	0.14	1.08	6.72	9.36
scyllo-Inositol, penta-TMS	N/A	2036	2038	trace	trae	-	-
Palmitelaidic acid, TMS	82326-15-6	2036	2038	-	-	0.11	-
Gluconic acid, hexa-TMS	34290-52-3	2045	2045	0.21	4.05	2.63	2.83
Palmitic acid, TMS	55520-89-3	2052	2052	0.15	0.16	0.79	1.35
7-Methyluric acid, tri-TMS	N/A	2124	2124	-	-	trace	0.12
myo-Inositol, hexa-TMS	2582-79-8	2129	2128	0.85	0.44	0.31	0.21
Oleic alcohol, mono-TMS	78695-25-7	2132	2136	-	0.04	-	-
Uric acid, tetraTMS	N/A	2144	2140	-	-	0.40	0.90
N-Acetylglucosamine, tetra-TMS?	31980-72-0	2150	2139	-	-	-	0.15
Heptadecanoic acid, TMS	55517-58-3	2150	2151	-	-	0.11	-
1-Octadecanol, TMS	18748-98-6	2165	2164	-	-	0.11	-
Isoproterenol, tri-TMS? (355,73,356…412)	29522-12-1	2174	-	-	0.04	-	-
NN (70,269,73,75,284…429)	-	2182	-	0.57	0.06	-	0.12
Noradrenaline, penta-TMS	56114-59-1	2190	-	-	0.72	-	-
Dopamine, tetra-TMS	N/A	2213	-	-	0.07	-	-
Linoleic acid, mono-TMS	56259-07-5	2215	2215	-	-	0.98	0.83
Oleic acid, mono-TMS	21556-26-3	2222	2222	0.42	0.12	5.63	7.05
Tryptamine, tri-N,N,N'-TMS?	55334-17-3	2234	-	-	0.03	0.42	-
Stearic acid, mono-TMS	18748-91-9	2248	2250	trace	0.09	1.48	1.46
myo-Inositol phosphate	55518-06-4	2259	2259	-	-	0.32	0.72
Glucoside, TMS	-	2261	-	-	-	-	0.22
Glucoside, TMS	-	2266	-	-	-	-	0.24
Glucoside, TMS	-	2270	-	-	-	-	0.18
NN	-	2271	-	-	-	-	0.15
Phosphate, TMS	-	2288	-	-	-	-	0.14
Carbohydrate derivative, TMS	-	2290	-	-	-	-	0.17
Carbohydrate derivative, TMS	-	2295	-	-	-	-	0.23
n-Tricosane	638-67-5	2300	2300	0.12	-	0.11	-
Carbohydrate derivative, TMS	-	2327	-	-	-	0.17	0.14
Eicosen-1-ol <(Z)>, mono-TMS	N/A	2334	2337	3.14	0.97	1.82	3.34
1-Eicosanol, TMS	N/A	2361	2360	-	-	0.34	0.18
NN (404)	-	2372	-	-	-	0.21	0.26
2-*O*-Glycerol-α-*D*-galactopyranoside, hexa-TMS	N/A	2376	2376	-	0.02	-	-
Pseudouridine, penta-TMS	53294-25-0	2388	-	-	0.04	-	-

Carbohydrate derivative, TMS	-	2388	-	-	-	0.16	0.17
NN (418>73,419,147)	-	2390		-	-	0.16	0.15
NN (217,73,147,257,259…475) uridine-like	-	2428	-	-	0.32	-	
NN	-	2428	-	0.01	-	0.13	0.48
Eicosanoic acid, TMS	55530-70-6	2449	2448	-	-	0.05	trace
Uridine, tri-TMS	10457-16-6	2472	2470	0.99	0.32	-	-
9-Pentacosene	N/A	2475	2470	-	-	0.04	-
Serotonin, tetra-TMS	55429-74-8	2486	-	-	0.04	-	-
n-Pentacosane	629-99-2	2500	2500	0.12	0.05	0.09	-
1-Docosanol, TMS	41755-60-6	2558	2557	-	-	0.06	trace
Galactitol derivative (73,319,204,205,217,307)	-	2585	-	-	0.04	-	-
Carbohydrate derivative, TMS		2605		-	-	-	0.12
Inosine riboside, tetra-TMS	N/A	2613	2614	1.91	0.80	-	0.56
Carbohydrate derivative, TMS		2626		-	-	0.09	0.15
Docosanoic acid, TMS	74367-36-5	2644	2645	-	-	0.10	trace
NN (73,147,515,217,425,630)	-	2656	-	0.25	-	0.13	-
NN	-	2690		-	-	0.36	0.15
NN	-	2699		-	-	-	0.14
n-Heptacosane	593-49-7	2700	2700	0.69	0.09	0.23	-
Disaccharide, octa-TMS	-	2705	-	-	-	0.21	-
Sucrose, octa-TMS	19159-25-2	2719	2714	15.85	2.97	trace	-
NN (73,103,147,217,574,307)	-	2735	-	1.16	0.10		-
1-Tetracosanol, TMS	N/A	2755	2754	-	-	0.08	trace
Cellobiose, octa-TMS, isomer-1	56145-25-6	2762	2762	0.15	0.09	-	-
α-Glyceryl oleate, di-TMS	54284-47-8	2778	2779	0.08	-	-	0.23
Disaccharide (73,361,147,204,217)	-	2778	-	-	0.03	--	-
Disaccharide (73,361,147,204,217)	-	2783	-	-	0.04	-	-
NN (73,252,245,217,230…571) inosine-like	-	2805	-	0.38	0.45	-	-
α-Glyceryl stearate, di-TMS	1188-75-6	2808	2805	-	-	-	0.66
Trehalose, octa-TMS	42390-78-3	2814		2.91	1.52	0.26	1.02
Guanosine, penta-TMS	N/A	2829	-	0.16	0.04	-	-
Palatinose, octa-TMS	N/A	2838	2832	-	-	0.42	0.25
Leucrose, octa-TMS	N/A	2885	2885	-	0.04	-	-
1-Eicosylglycerol, di-TMS	N/A	2890	2884	0.62	0.03	-	0.08
Cellobiose,octa-TMS, isomer-2	-N/A	2894	-	0.17	-	0.21	-
n-Nonacosane	630-03-5	2900	2900	trace	trace	-	-
Disaccharide, TMS (217,204,73,147,103)	-	2942	-	0.08	0.06	0.07	-
1-Hexacosanol, TMS	N/A	2953	2951	-	-	0.10	
β-Isomaltose, octa-TMS	N/A	3007	3005	-	-	0.08	-
9-Triacontene	N/A	3076	3078	0.22	0.03	0.10	-
7-Triacontene	N/A	3083	3082	0.14	0.03	0.21	-
24-Methylenecholesterol, mono-TMS	N/A	3251	3244	-	-	1.33	1.08
7-Tritriacontene	N/A	3278	3278	0.39	0.10	-	
β-Sitosterol, mono-TMS	2625-46-9	3348	3345	-	-	0.16	0.14
Isofucosterol, mono-TMS	N/A	3364	3363	-	-	0.37	0.42
Palmitate	-	3422	-	-	-	0.91	-

1-Kestose, undeca-TMS	N/A	3518	3515	0.27	0.07	-	-
Erlose, undeca-TMS	N/A	3555	3549	1.26	0.07	-	-
Melizitose, undeca-TMS	N/A	3590	3588	0.77	trace	-	-
NN (m/z 255,129,73)	-	3735	-	-	-	0.15	-
NN (m/z 255,129,73)	-	3927	-	-	-	0.54	-
Oleate	-	3935	-	-	-	0.10	-
Palmitate	-	>4000	-	-	-	0.99	-
